# Oral-to-rectum microbial transmission in orthopedic patients without a history of intestinal disorders

**DOI:** 10.3389/fcimb.2024.1358684

**Published:** 2024-04-10

**Authors:** Ge Lin, Shinya Kageyama, Aiko Maeda, Eiji Sakamoto, Jiale Ma, Mikari Asakawa, Michiko Furuta, Yoshihisa Yamashita, Toru Takeshita

**Affiliations:** ^1^ Section of Preventive and Public Health Dentistry, Division of Oral Health, Growth and Development, Faculty of Dental Science, Kyushu University, Fukuoka, Japan; ^2^ Department of Anesthesiology and Critical Care Medicine, Kyushu University Hospital, Fukuoka, Japan; ^3^ Department of Oral and Maxillofacial Surgery, Kyushu University Hospital, Fukuoka, Japan

**Keywords:** tongue, gut, microbiota, translocation, 16S rRNA, aging, hypertension, proton pump inhibitor

## Abstract

The enrichment of oral taxa in the gut has recently been reported as a notable alteration in the microbial balance in patients with intestinal disorders. However, translocation in populations without such diseases remains controversial. In this study, we examined 49 pairs of tongue and rectal samples collected from orthopedic patients without a history of intestinal disorders to verify the presence of oral taxa in the rectal microbiota. The bacterial composition of each sample was determined using 16S rRNA gene sequencing and amplicon sequence variant (ASV) analysis. Although the bacterial compositions of the tongue and rectal microbiota were distinctly different, tongue ASVs were detected in 67.3% of the participants and accounted for 0.0%–9.37% of the rectal microbiota. Particularly, *Streptococcus salivarius*, *Fusobacterium nucleatum*, and *Streptococcus parasanguinis* were abundant in the rectal microbiota. According to the network analysis, tongue taxa, such as *S. salivarius* and *S. parasanguinis*, formed a cohabiting group with *Klebsiella pneumoniae* and *Alistipes finegoldii* in the rectal microbiota. The total abundance of tongue ASVs in the rectal microbiota was significantly higher in participants with older age, hypertension, and proton pump inhibitor (PPI) use. Our study presents an extensive translocation of oral taxa to the rectum of a population without intestinal disorders and suggests that aging, hypertension, and PPI use are associated with an increased abundance of oral taxa and potential pathogenic bacteria in the rectal microbiota.

## 1 Introduction

Numerous microbes inhabit the human intestine forming diverse and unique microbial communities (Huttenhower et al., 2012; Segata et al., 2012). They play a crucial role in preserving the physiological homeostasis of the host by making essential contributions to the host metabolism and immune system (Round and Mazmanian, 2009; Nicholson et al., 2012). Consistent with this notion, dysbiosis of gut microbiota has been reported to be involved in intestinal disorders such as inflammatory bowel disease (IBD) (Franzosa et al., 2019; Lavelle and Sokol, 2020) and colorectal cancer (CRC) (Wong and Yu, 2019). Remarkably, the abnormal enrichment of oral bacteria has been observed in the guts of patients with intestinal diseases (Strauss et al., 2011; Atarashi et al., 2017; Yachida et al., 2019; Kitamoto et al., 2020a). For instance, *Fusobacterium nucleatum*, which generally inhabits the oral cavity, is frequently detected in patients with IBD and CRC (Strauss et al., 2011; Yachida et al., 2019). Although the causal association between oral bacterial enrichment and intestinal diseases remains unclear, the possible ectopic colonization of oral bacteria in the gut plays a significant role in the etiology of intestinal diseases. In fact, studies have indicated that intestinal colonization of *Klebsiella pneumoniae* originating from the saliva of patients with IBD induces gut inflammation in mice (Atarashi et al., 2017).

The oral cavity, the gateway of the digestive tract, hosts a large microbial community distinct from the gut microbiota (Huttenhower et al., 2012; Segata et al., 2012). People constantly swallow such copious amounts of oral microbes with saliva and boluses of food (Humphrey and Williamson, 2001). Considering the antimicrobial barriers along the gastrointestinal tract, such as gastric acidity (Giannella et al., 1972), bile (Begley et al., 2005), and colonization resistance by the gut-resident microbiota, ingested oral bacteria are unlikely to reach and colonize the gut in healthy individuals. In line with this, a previous study identified no evidence of colonization by oral bacteria in the guts of healthy adults (Rashidi et al., 2021). However, metagenomic approaches or full-length 16S rRNA gene sequencing have demonstrated extensive translocation of oral bacteria to the gut in healthy individuals or community-dwelling adults (Schmidt et al., 2019; Kageyama et al., 2023); thus, the argument as to whether oral bacteria can be translocated to the gut remains inconclusive, especially in populations without specific intestinal disorders. To understand the causal association between oral bacteria and intestinal disorders, or their utility as predictors, verification of oral bacterial translocation to the gut in such populations is required.

In this study, we examined paired tongue and rectal microbiota from orthopedic patients without a history of intestinal disorders using 16S rRNA gene amplicon sequencing and the amplicon sequence variant (ASV) approach. Instead of stool samples which are usually used for gut microbiota analysis, rectal swab samples were collected. The bacterial community on the mucosal surface of the rectum is known to display a composition different from that of the stool (Zmora et al., 2018), and rectal swab samples can be used to examine the microbial profile on the mucosal surface of the rectum. This approach enables the evaluation of oral bacterial translocation to the gut from a novel perspective and could provide robust evidence of the translocation. The ASV approach resolves differences of as little as one nucleotide and enhances taxonomic resolution (Callahan et al., 2016). This study aimed to verify oral bacterial detection in the rectal microbiota at the ASV level and identify the relevant factors influencing the increased abundance of oral bacteria in the rectum.

## 2 Materials and methods

### 2.1 Study participants

The study participants were orthopedic patients who underwent surgery under general anesthesia at Kyushu University Hospital in Japan. To non-invasively examine rectal microbiota using rectal temperature probes, we only included patients who underwent surgery under general anesthesia. Among them, orthopedic patients were selected because they were less likely to have a history of intestinal disorders. We enrolled a total of 54 patients aged ≥20 years without a history of intestinal disorders and antibiotic use within 3 months. After excluding five participants whose samples were insufficient, 49 patients were finally included in the analysis. These patients most commonly had lumbar spinal canal stenosis (36.7%), followed by cervical spondylotic myelopathy (12.2%), osteonecrosis of the femoral head (12.2%), osteoarthritis of the hip (10.2%), and acetabular dysplasia (6.1%). Written informed consent was obtained from all participants. The Ethics Committee of Kyushu University approved the present study and the procedure for obtaining informed consent (approval number: 21165-01).

### 2.2 Sample collection and health information

Tongue swab samples were collected by scraping the dorsum of the tongue with a Puritan Hydraflock swab (Puritan Medical Products, Guilford, ME, USA) preoperatively immediately before surgery. After surgery, the rectal temperature probe was collected by cutting the inserted head of the probe as a rectal swab sample. Samples were transported to our laboratory and stored at -80°C until further analysis. Health information including medication history, body mass index, smoking habits, and alcohol intake was obtained from the electronic health record system of Kyushu University Hospital. Hypertension and diabetes were defined as the current use of antihypertensive and antidiabetic agents, respectively. In addition, we recorded the current use of steroids, nonsteroidal anti-inflammatory drugs (NSAIDs), opioids, proton pump inhibitors (PPIs), and immunosuppressants. Smoking habits and alcohol intake were categorized as current or non-current use.

### 2.3 16S rRNA gene amplicon sequencing

DNA was similarly extracted from the tongue and rectal samples using the bead-beating method described previously (Kageyama et al., 2022) after the swab or probe was discarded following centrifugation. The V1–V2 regions of the 16S rRNA gene were targeted for sequencing (Wade and Prosdocimi, 2020; Kameoka et al., 2021), and amplified using the following primers with the sample-specific eight-base tag sequence: 8F (5′-AGA GTT TGA TYM TGG CTC AG-3′) and 338R (5′-TGC TGC CTC CCG TAG GAG T-3′). PCR amplification was carried out using KOD DNA polymerase (Toyobo, Osaka, Japan) under the following cycling conditions: 98°C for 2 min, followed by 30 cycles of 98°C for 15 s, 60°C for 20 s, and 74°C for 30 s. Each PCR amplicon was purified using an Agencourt AMPure XP kit (Beckman Coulter, Brea, CA, USA), and equal amounts of purified amplicons were pooled. Pooled DNA was gel-purified using a Wizard SV Gel and PCR Clean-Up System (Promega, Madison, WI, USA). DNA concentration was determined using the KAPA Library Quantification Kit (KAPA Biosystems, MA, USA). Emulsion PCR and enrichment of template-positive particles were performed using the Ion 520 and Ion 530 Kit-OT2 (Thermo Fisher Scientific) on an Ion One Touch 2 system (Thermo Fisher Scientific). The enriched particles were loaded onto an Ion 530 Chip (Thermo Fisher Scientific), and sequencing was performed on the Ion GeneStudio S5 System (Thermo Fisher Scientific) using Ion 520 and Ion 530 Kit-OT2 (Thermo Fisher Scientific).

### 2.4 Data processing and taxonomy assignment

Raw sequence reads were excluded when they exhibited <100 bases or did not include the correct forward and reverse primer sequences using R software (version 4.2.3). The remaining reads were demultiplexed by examining the eight-base tag sequence at both ends, and the forward and reverse primer sequences were trimmed. The reads were further processed using the DADA2 pipeline (version 1.26.0) software, including quality-filtering, denoising, and chimera-filtering procedures with default settings for Ion Torrent sequences, and an ASV table was produced (Callahan et al., 2016). Five ASVs corresponding to *Pseudomonas fluorescens* HMT-612, predominantly observed in the negative control, were excluded from the ASV table and subsequent analysis as PCR contaminants. The genus-level taxonomy of each sequence variant was determined using the RDP classifier with a minimum support threshold of 80% and RDP taxonomic nomenclature (Wang et al., 2007). Species-level taxonomic assignment was performed using BLAST (Altschul et al., 1990) against 1,015 oral bacterial 16S rRNA gene sequences (16S rRNA RefSeq version 15.22) in expanded Human Oral Microbiome Database (eHOMD) (Chen et al., 2010). Nearest-neighbor species with ≥98.5% identity were selected as candidates for each sequence. For sequences without hits against eHOMD, the species-level taxonomy was further determined using the assignTaxonomy function in DADA2 with Silva 132 taxonomic training data (silva_nr_v132_train_set.fa.gz) (Quast et al., 2013; Yilmaz et al., 2014; Callahan et al., 2016).

### 2.5 Statistical analysis

All statistical analyses were performed using R software. Differences in bacterial composition between the salivary and rectal microbiota were evaluated using the Bray–Curtis distance based on the log-transformed ASV data. Bray–Curtis distances, the total relative abundance of tongue ASVs in the rectal microbiota, and the percentage of tongue ASVs over the total number of ASVs observed in the rectal microbiota were compared within an individual (intra-pair) and across different individuals (inter-pair) using the Wilcoxon signed–rank test. To evaluate the translocation of tongue bacteria to the rectum, we identified ASVs accounting for 0.1% of the tongue microbiota as tongue ASVs for each participant. Subsequently, we computed the overall abundance of tongue ASVs present in the rectal microbiota for each participant. The Kruskal–Wallis test, along with effect size calculation (η^2^), was employed to compare the overall abundance of tongue ASVs in the rectum across different clinical factors. Furthermore, the significance was calculated again using the Steel–Dwass test or the exact Wilcoxon rank sum test from the *exactRankTests* package. Co-occurrence network analysis was performed for 71 species with ≥0.1% of mean relative abundance in the rectal microbiota using the SparCC algorithm (Friedman and Alm, 2012) within the sparse co-occurrence network investigation for compositional data tool (Shaffer et al., 2023) in the QIIME 2 (Bolyen et al., 2019). Co-occurrence relationships with the SparCC R values of ≥0.4 were visualized as edges using the *sna* package.

## 3 Results

### 3.1 Characteristics of participants and 16S rRNA gene sequencing

We examined 49 pairs of tongue and rectal samples collected from orthopedic patients who underwent surgery. Detailed characteristics of the participants are presented in **Table 1**. Their median age was 63 years (range, 23–89 years). The prevalence rates of hypertension and diabetes were 51.0% and 14.3%, respectively. A total of 98 samples from 49 participants were analyzed using 16S rRNA gene amplicon analysis on an Ion GeneStudio S5 System. Finally, 3,262,022 denoised reads (30,323 ± 6,547 reads per tongue sample and 36,248 ± 11,175 reads per rectal sample) and 7,222 ASVs were obtained. Only 2,883 ASVs (40.0%) were assigned a species-level taxonomy, and they accounted for 92.9 ± 4.3% of tongue microbiota and 59.0 ± 15.5% of rectal microbiota.

**Table 1:** Characteristics of participants.

	n (%)
Male	25 (51.0)
Age (years)	
20–39	9 (18.4)
40–59	11 (22.4)
60–79	23 (46.9)
≥80	6 (12.2)
BMI	
<18.5	3 (6.1)
≥18.5 and <25	29 (59.2)
≥25	17 (34.7)
Alcohol intake	13 (26.5)
Current smoking	15 (30.6)
Hypertension	25 (51.0)
Diabetes	7 (14.3)
Medications	
Steroid	6 (12.2)
NSAID	16 (32.7)
Opioid	3 (6.1)
Proton pump inhibitor	12 (24.5)
Immunosuppressant	6 (12.2)

BMI, body mass index; NSAID, nonsteroidal anti-inflammatory drugs.

### 3.2 Microbial difference between tongue and rectal microbiota

**Figure 1:**
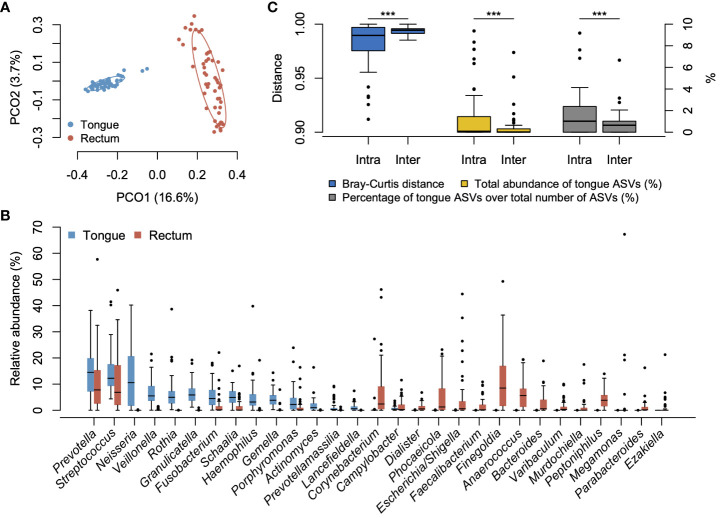
Bacterial compositions of tongue and rectal microbiota. **(A)** Principal coordinate analysis plot based on the Bray–Curtis distance at the ASV level. The bacterial composition of tongue and rectal samples is depicted using different colors. The ellipse covers 67% of the samples belonging to each group. **(B)** Compositional difference between tongue and rectal microbiota. Twenty-nine genera with ≥1% of mean relative abundance in either tongue or rectal microbiota are displayed. **(C)** Compositional similarity between the tongue and rectal microbiota, the total relative abundance of tongue ASVs in the rectal microbiota, and the percentage of tongue ASVs over the total number of ASVs observed in the rectal microbiota within an individual (intra-pair) and across different individuals (inter-pair). ***P <0.001.

We compared the overall bacterial composition of the tongue and rectal microbiota. According to the principal coordinate analysis plot based on the Bray–Curtis distance at the ASV level, the bacterial compositions in the tongue and rectum exhibited marked distinctions (**Figure 1A**). In the tongue microbiota, 15 genera showed ≥1% of mean relative abundance and *Prevotella*, *Streptococcus*, and *Neisseria* were particularly predominant (**Figure 1B**). At the species level, *Neisseria perflava* HMT-101, *Rothia mucilaginosa* HMT-681, *Prevotella melaninogenica* HMT-469, *Granulicatella adiacens* HMT-534, and *Hemophilus parainfluenzae* HMT-718 were the predominant species (**Supplementary Figure S1**). The rectal microbiota was dominated by 20 genera including *Prevotella*, *Finegoldia*, and *Streptococcus*. *Finegoldia magna* HMT-662, *Streptococcus anginosus* HMT-543, *Enterobacter hormaechei* HMT-634, *Prevotella bivia* HMT-556, and *Corynebacterium simulans* HMT-062 were predominantly detected (**Supplementary Figure S1**).

### 3.3 Translocation of tongue bacteria in rectal microbiota

**Figure 2:**
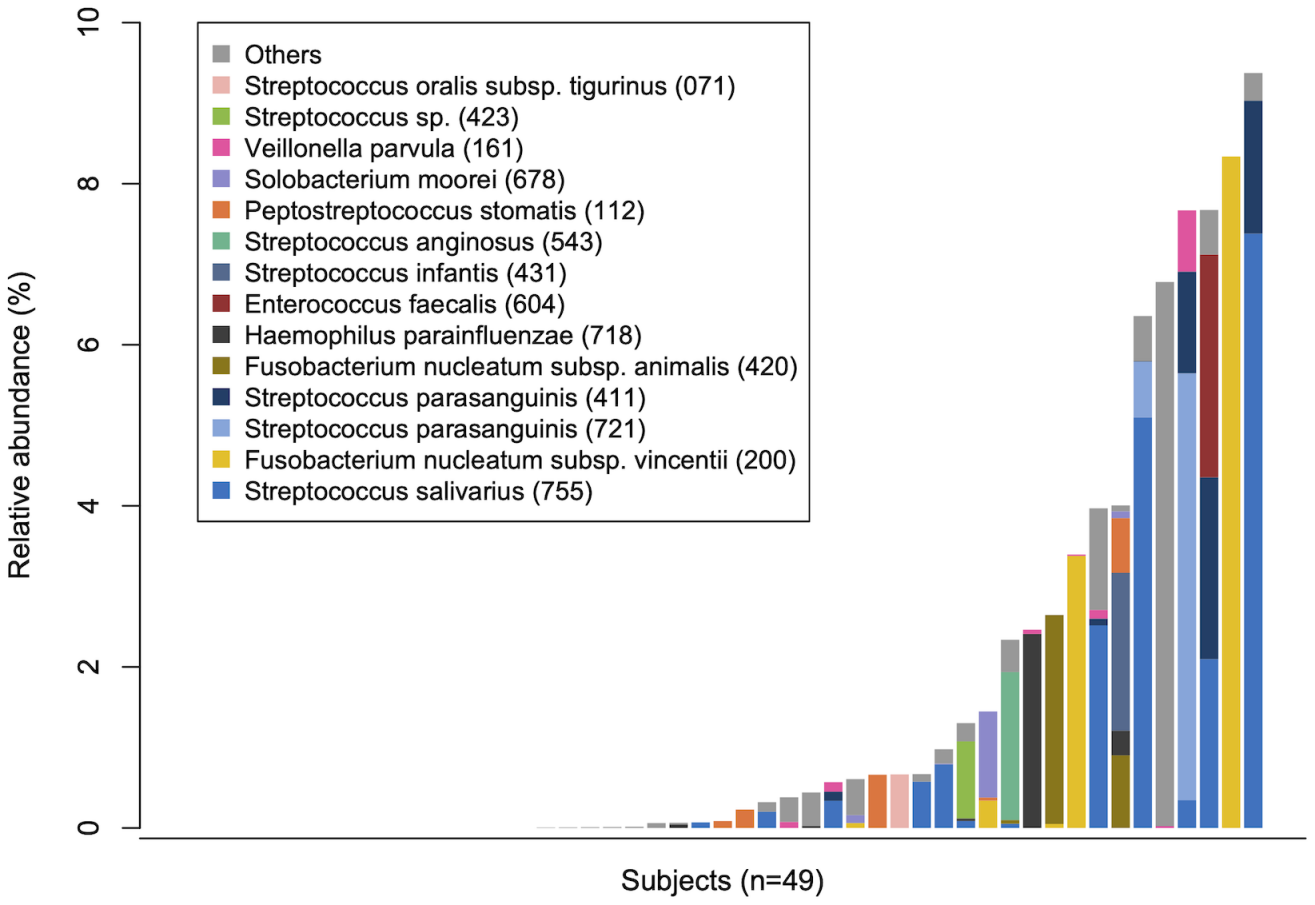
Tongue bacteria in the rectal microbiota. The total relative abundance of tongue amplicon sequence variants (ASVs) in rectal microbiota in each participant is demonstrated in ascending order. The composition is displayed at the species level. Bacterial species with ≥0.5% of mean relative abundance in the rectal microbiota are depicted by different colors. Human microbial taxon numbers in the expanded Human Oral Microbiome Database (eHOMD) are provided in parentheses following bacterial names.

To evaluate the translocation of tongue bacteria to the rectum in each participant, we investigated the microbial sharing between the tongue and the rectal microbiota within an individual (intra-pair) and across different individuals (inter-pair). The similarity of bacterial composition in intra-pairs was significantly higher than that in inter-pairs (**Figure 1C**). The total relative abundance of tongue ASVs in the rectal microbiota, and the percentage of tongue ASVs over the total number of ASVs observed in the rectal microbiota were also significantly higher in intra-pairs than in inter-pairs (**Figure 1C**). Tongue ASVs were detected in 33 participants (67.3%) and accounted for 0.0%–9.37% of the rectal microbiota (**Figure 2**). In particular, *Streptococcus salivarius* HMT-755, *F. nucleatum* subspecies *vincentii* HMT-200, *Streptococcus parasanguinis* HMT-721 and HMT-411, and *F. nucleatum* subspecies *animalis* HMT-420 were abundant in the rectal microbiota.

### 3.4 Co-occurrence network in the rectal microbiota

**Figure 3:**
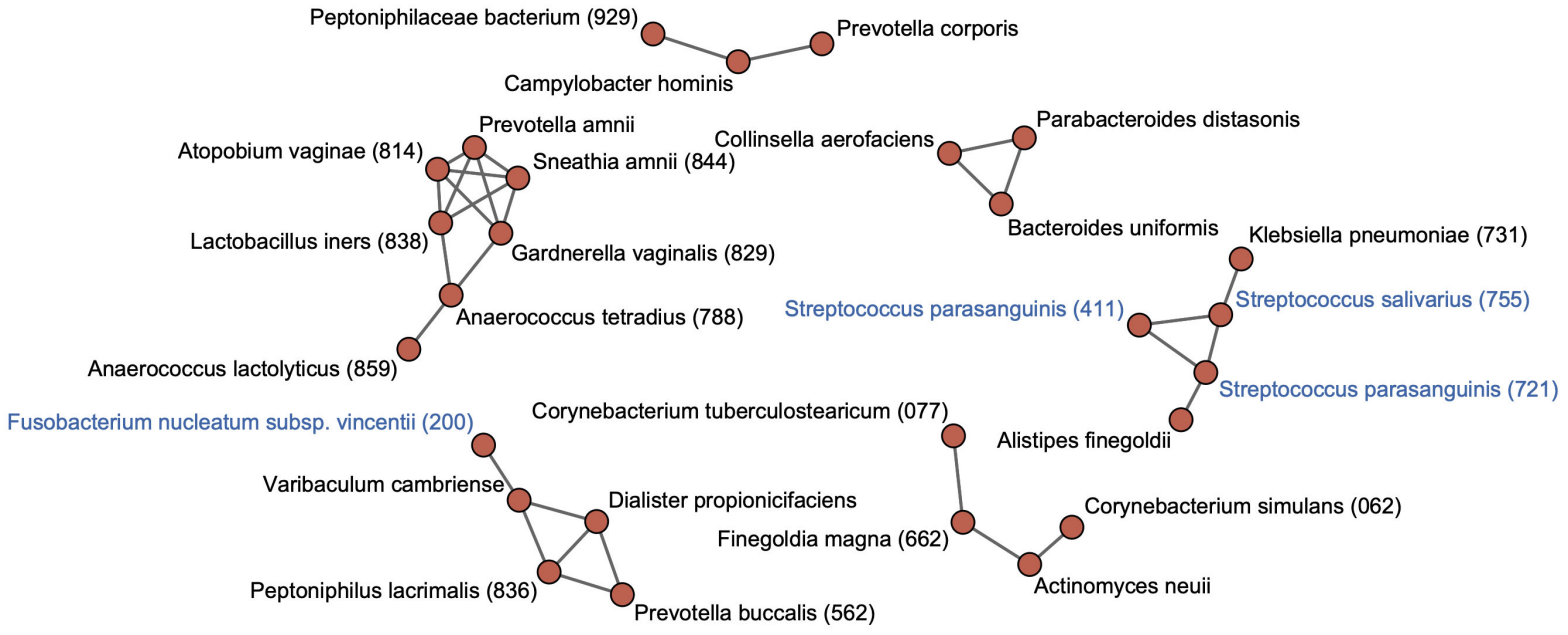
Co-occurrence network in rectal bacterial species. Co-occurrence network analysis is performed for 71 species with ≥0.1% of mean relative abundance in the rectal microbiota. Co-occurrence relationships with the SparCC R values of ≥0.4 are visualized as edges. Bacterial species with ≥0.1% of mean relative abundance in the tongue microbiota are indicated by blue color. Human microbial taxon (HMT) numbers in the expanded Human Oral Microbiome Database (eHOMD) are provided in parentheses following bacterial names. Bacterial names without HMT numbers are assigned based on the SILVA database.

To identify cohabiting groups in the rectal microbiota, we performed a co-occurrence network analysis for predominant species with ≥0.1% of mean relative abundance in the rectal microbiota. This approach identified six cohabiting groups which comprised the predominant species in the rectal microbiota; the most abundant group was composed of *Finegoldia magna* HMT-662, *Corynebacterium simulans* HMT-062, *Corynebacterium tuberculostearicum* HMT-077, and *Actinomyces neuii* (**Figure 3**). Tongue taxa such as *S. salivarius* and *S. parasanguinis* formed one group with *K. pneumoniae* HMT-731 and *Alistipes finegoldii*. The *F. nucleatum* subspecies *vincentii* was found to belong to a different cohabiting group than the group that included the tongue taxa, and exhibited co-occurrence with other members of the rectal microbiota.

### 3.5 Influential factors on the increased abundance of tongue bacteria in the rectal microbiota

**Figure 4:**
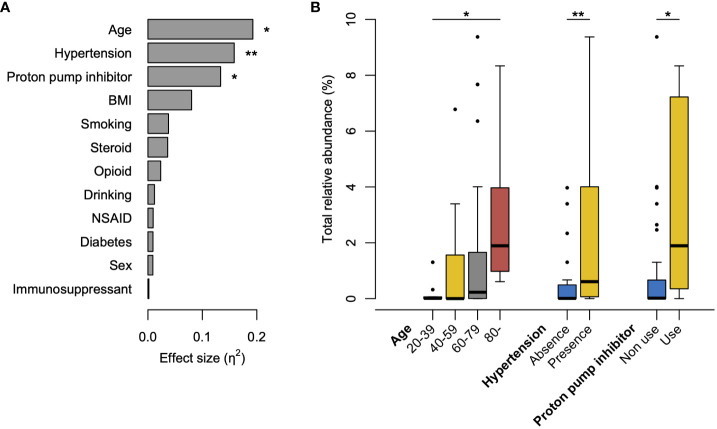
Factors influencing the increased abundance of tongue bacteria in the rectal microbiota. **(A)** Effects of clinical factors on the total relative abundance of tongue amplicon sequence variants (ASVs) in rectal microbiota. The effect size (η^2^) and the significance are respectively calculated using the Kruskal–Wallis test. **P <0.01, *P <0.05. **(B)** Boxplot of the total abundance of tongue ASVs in rectal microbiota according to the factors with significance in the Kruskal–Wallis test. The significance is calculated using the Steel–Dwass test or the exact Wilcoxon rank sum test. **P <0.01; *P <0.05. BMI, body mass index; NSAID, nonsteroidal anti-inflammatory drugs.

To explore the clinical factors affecting the increased abundance of tongue bacteria in the rectum, we compared the occupancy of tongue ASVs in the rectal microbiota according to the clinical characteristics. The Kruskal–Wallis test demonstrated that age, presence of hypertension, and use of PPI were significantly associated with the total relative abundance of tongue ASVs in the rectum (**Figure 4A**). The abundance demonstrated an increasing trend with age and was significantly higher in participants aged ≥80 years than in participants aged 20–39 years (**Figure 4B**). Participants with hypertension or PPI use had a higher abundance of tongue ASVs in the rectum compared to those without hypertension or those not using PPIs.

## 4 Discussion

This study examined 49 pairs of tongue and rectal samples collected from orthopedic patients without a history of intestinal disorders using 16S rRNA gene amplicon analysis and the ASV approach, and verified the detection of own oral bacteria in the rectum. The tongue ASVs were detected from the rectal microbiota in 67.3% of participants and demonstrated ≥1% relative abundance in 28.6% of participants. Further, the bacterial composition of the rectum was significantly more similar to the individual’s own tongue than to others’ tongue. These results suggest that oral bacteria can endogenously reach the rectum, transverse the digestive tract, and colonize the mucosal surface, even in the absence of intestinal disorders. This observation supports the findings of a previous study that suggested the oral gut transmission of microbes in healthy participants or community-dwelling adults (Schmidt et al., 2019; Kageyama et al., 2023). Oral bacterial translocation can occur extensively, and not only in populations with specific diseases.

Oral taxa such as *S. salivarius*, *S. parasanguinis*, and *F. nucleatum* were abundantly detected in the rectal microbiota. As stated in the Introduction, *F. nucleatum* is known to be enriched in the gut of patients with IBD and CRC and is suggested to induce intestinal inflammation and tumorigenesis (Strauss et al., 2011; Kostic et al., 2013; Yachida et al., 2019). Interestingly, this study also detected *Solobacterium moorei* HMT-678 and *Peptostreptococcus stomatis* HMT-112 in the rectal microbiota. Their abundances have been reported to increase in the early stages of CRC, similar to that of *F. nucleatum* (Yachida et al., 2019). Furthermore, network analysis displayed the co-occurrence of *S. salivarius* and *S. parasanguinis* with *K. pneumoniae*, which is reported to induce gut inflammation in mice (Atarashi et al., 2017; Kitamoto et al., 2020b), in the rectum, consistent with our previous study examining salivary and stool samples (Kageyama et al., 2023). Thus, the findings of this study are highly consistent with those of previous reports examining oral gut transmission. Importantly, these findings were observed in a population with no history of intestinal disorders. The observations above suggest that oral bacterial translocation is associated with rectal dysbiosis, characterized by an increase in potentially pathogenic bacteria, which could begin when people are healthy.

Aging was significantly associated with an increased abundance of tongue ASVs in the rectal microbiota (**Figure 4**). This could be attributed to a diminished barrier function in the gastrointestinal tract with advancing age (Drozdowski and Thomson, 2006), consistent with the results of our previous study (Kageyama et al., 2023). Although distinguishing between pathological alterations and age-induced changes is difficult, the translocation of oral bacteria to the gut can be considered an age-related change that occurs widely in older individuals. Furthermore, considering the enrichment of potential carcinogenic bacteria, this age-related change in the gut microbiota might partially contribute to the high incidence of CRC in older adults. Similarly, PPI use, which reduces gastric acid secretion, contributed to an increase in oral bacteria in the rectum (**Figure 4**), as some studies using stool samples previously reported (Imhann et al., 2016; Matthew et al., 2016; Jiaying et al., 2023). PPI use is also a known risk factor for *Clostridium difficile* infection, which is characterized by various gastrointestinal symptoms (Kwok et al., 2012; Inghammar et al., 2021). In this study, the prevalence was not statistically significant; however, *C. difficile* was only observed in the rectum of PPI users (n=1). Interestingly, the participants with hypertension exhibited a significantly high abundance of oral bacteria in the rectal microbiota (**Figure 4**). A recent study reported that the transplantation of saliva from individuals with hypertension via oral gavage exacerbated angiotensin II-induced hypertension in mice (Chen et al., 2023). The causal association between hypertension and translocation of oral bacteria should be carefully considered in future studies.

This study has several limitations. First, oral health information such as dental caries, periodontal condition, number of teeth present, and oral hygiene was not obtained. Our previous study suggested that dental plaque accumulation was associated with an increased abundance of oral bacteria in the gut (Kageyama et al., 2023). Therefore, the influence of oral health conditions on oral bacterial occupancy in the rectum should be further considered. Second, our analyses relied on tongue microbiota, despite the fact that oral bacteria are likely transported to the gut via saliva. However, previous studies have reported that the salivary microbiota is mainly composed of tongue microbes (Segata et al., 2012; Kageyama et al., 2017). Meanwhile, further consideration should be given to the translocation of dental plaque-preferring bacteria such as *F. nucleatum* because the bacterial composition on the tongue is different from that on dental plaque. The translocation of dental plaque-preferring bacteria may have been underestimated in this study. Third, species-level taxonomy was assigned to only an average of 59.0% of each rectal microbiota, unlike 92.9% for tongue microbiota based on a well-developed database for oral taxa, HOMD (Chen et al., 2010). Thus, network analysis of rectal bacterial species may not detect or underestimate the co-occurrence network. Fourth, all the participants were administered antibiotics during surgery. However, we consider that the effect of antibiotics on the bacterial composition of the rectal microbiota examined in this study was minimal because the rectal sample was collected immediately after surgery and the outcome of the 16S rRNA gene analysis is typically independent of whether the bacteria are alive or dead. On the contrary, the fifth limitation is that the 16S rRNA gene analysis could not evaluate whether the tongue bacteria in the rectum were alive or dead. However, considering their dominance in the rectum, they were likely to thrive. A previous study using a culture approach demonstrated that *F. nucleatum* strains identical to the oral strains were isolated alive from the gut (Komiya et al., 2019). Finally, short-read sequencing of the 16S rRNA gene did not reveal differences between conspecific strains, and the translocation of oral bacteria may have been overestimated. Further studies that utilize long-read sequencing or metagenomic approaches are now required to validate our results.

In conclusion, this study examined tongue and rectal samples using 16S rRNA gene amplicon analysis and the ASV approach and demonstrated the translocation of tongue bacteria to the rectum based on ASV sharing (i.e., identical match of the V1–V2 region of the 16S rRNA gene). Furthermore, this detection was observed in 67.3% of participants without intestinal disorders. These results suggested that oral bacterial translocation occurs extensively in healthy individuals. Moreover, translocation was involved in the increased abundance of potentially pathogenic bacteria, suggesting that translocation might play a role in the initial events of intestinal disorders. Monitoring oral bacteria in the gut could be useful for evaluating intestinal disease risk. These findings could contribute to the development of novel preventive strategies against intestinal diseases by inhibiting the translocation of oral bacteria. Further studies are needed to determine the association between the translocation of oral bacteria and the subsequent risk of disease.

## Data availability statement

The sequence data presented in the study are deposited in the DDBJ BioProject database, accession number PRJDB17727.

## Ethics statement

The studies involving humans were approved by The Ethics Committee of Kyushu University. The studies were conducted in accordance with the local legislation and institutional requirements. The participants provided their written informed consent to participate in this study.

## Author contributions

GL: Investigation, Visualization, Writing – original draft. SK: Conceptualization, Funding acquisition, Visualization, Writing – original draft, Writing – review & editing. AM: Conceptualization, Investigation, Writing – review & editing. ES: Conceptualization, Investigation, Writing – review & editing. JM: Investigation, Writing – review & editing. MA: Investigation, Writing – review & editing. MF: Supervision, Writing – review & editing. YY: Conceptualization, Supervision, Writing – review & editing. TT: Funding acquisition, Supervision, Writing – review & editing.

## Supplementary material

Supplementary Figure 1 Predominant bacterial species in tongue and rectal microbiota. Thirty-two species with ≥1% of mean relative abundance in either tongue or rectal microbiota are displayed in descending order of their mean relative abundance in tongue microbiota. Human microbial taxon (HMT) numbers in the expanded Human Oral Microbiome Database (eHOMD) are provided in parentheses following bacterial names. Bacterial names without HMT numbers are assigned based on the SILVA database.
