# Supplementary figures and images for: Oral-to-rectum microbial transmission in orthopedic patients without a history of intestinal disorders
Source: Front Cell Infect Microbiol. 2024 Apr 10;14:1358684. doi: 10.3389/fcimb.2024.1358684 (PMC11039792; doi:10.3389/fcimb.2024.1358684)

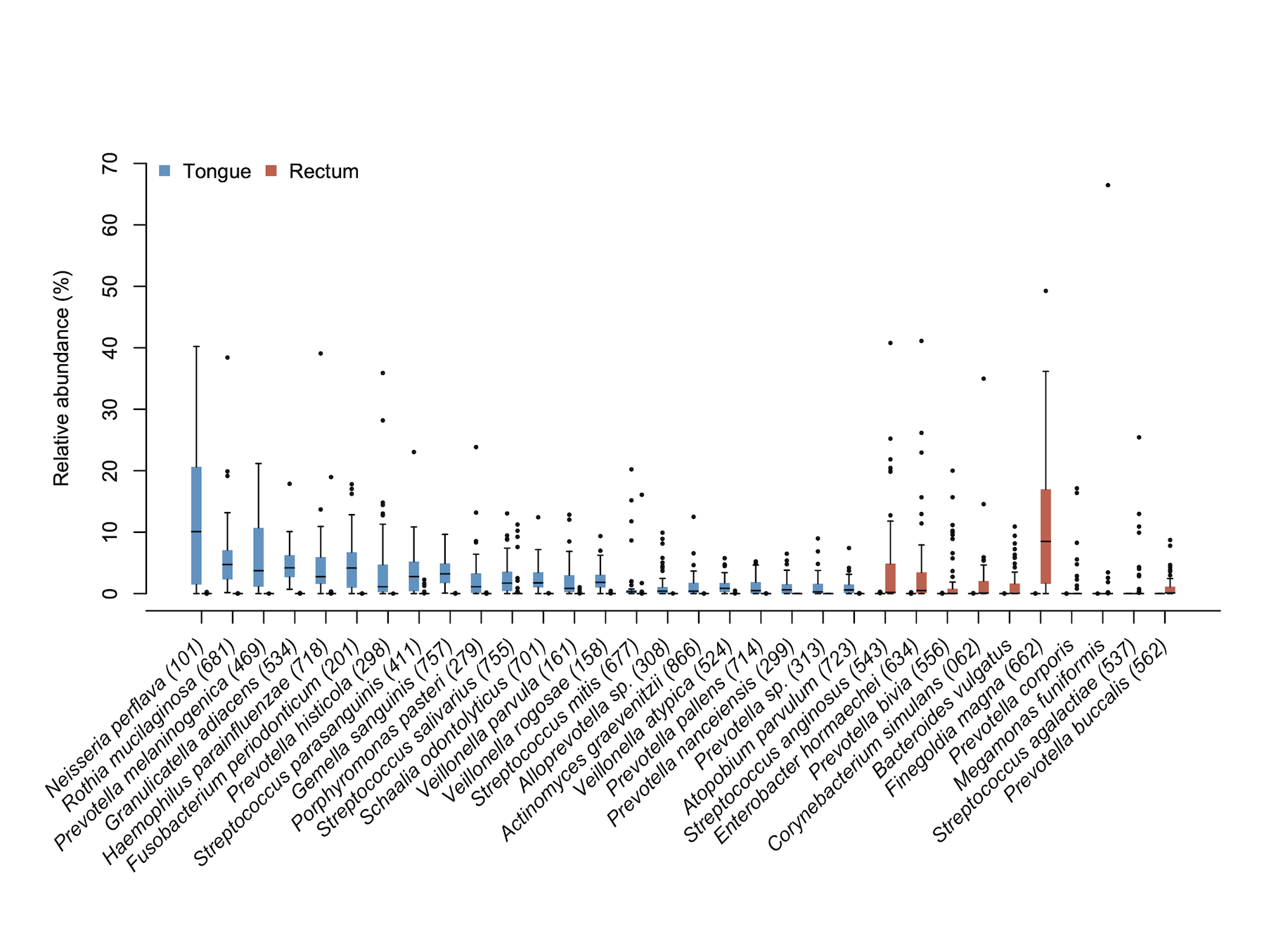

Supplement: Supplementary Figure 1 — Predominant bacterial species in tongue and rectal microbiota. Thirty-two species with ≥1% of mean relative abundance in either tongue or rectal microbiota are displayed in descending order of their mean relative abundance in tongue microbiota. Human microbial taxon (HMT) numbers in the expanded Human Oral Microbiome Database (eHOMD) are provided in parentheses following bacterial names. Bacterial names without HMT numbers are assigned based on the SILVA database. [file Image_1.tiff]
